# Adult‐type granulosa cell tumor of the ovary: a *FOXL2*‐centric disease

**DOI:** 10.1002/cjp2.198

**Published:** 2021-01-11

**Authors:** Jessica A Pilsworth, Dawn R Cochrane, Samantha J Neilson, Bahar H Moussavi, Daniel Lai, Aslı D Munzur, Janine Senz, Yi Kan Wang, Sina Zareian, Ali Bashashati, Adele Wong, Jacqueline Keul, Annette Staebler, Hannah S van Meurs, Hugo M Horlings, Stefan Kommoss, Friedrich Kommoss, Esther Oliva, Anniina EM Färkkilä, Blake Gilks, David G Huntsman

**Affiliations:** ^1^ Department of Molecular Oncology British Columbia Cancer Research Centre Vancouver BC Canada; ^2^ Department of Medical Genetics University of British Columbia Vancouver BC Canada; ^3^ Department of Pathology and Laboratory Medicine University of British Columbia Vancouver BC Canada; ^4^ School of Biomedical Engineering University of British Columbia Vancouver BC Canada; ^5^ Department of Pathology Massachusetts General Hospital Boston MA USA; ^6^ Department of Women's Health Tübingen University Hospital Tübingen Germany; ^7^ Institute of Pathology and Neuropathology Tübingen University Hospital Tübingen Germany; ^8^ Department of Gynecology Center for Gynecologic Oncology Amsterdam, Academic Medical Center Amsterdam The Netherlands; ^9^ Department of Pathology The Netherlands Cancer Institute – Antoni van Leeuwenhoek Amsterdam The Netherlands; ^10^ Institute of Pathology, Medizin Campus Bodensee Friedrichshafen Germany; ^11^ Research Program for Systems Oncology University of Helsinki and Helsinki University Hospital Helsinki Finland

**Keywords:** adult‐type granulosa cell tumor of the ovary, *FOXL2*, *TERT* promoter, *KMT2D*, targeted sequencing, mutation profiling, cell cycle genes, sex cord‐stromal tumor, ovarian cancer

## Abstract

Adult‐type granulosa cell tumors (aGCTs) account for 90% of malignant ovarian sex cord‐stromal tumors and 2–5% of all ovarian cancers. These tumors are usually diagnosed at an early stage and are treated with surgery. However, one‐third of patients relapse between 4 and 8 years after initial diagnosis, and there are currently no effective treatments other than surgery for these relapsed patients. As the majority of aGCTs (>95%) harbor a somatic mutation in *FOXL2* (c.C402G; p.C134W), the aim of this study was to identify genetic mutations besides *FOXL2* C402G in aGCTs that could explain the clinical diversity of this disease. Whole‐genome sequencing of 10 aGCTs and their matched normal blood was performed to identify somatic mutations. From this analysis, a custom amplicon‐based panel was designed to sequence 39 genes of interest in a validation cohort of 83 aGCTs collected internationally. *KMT2D* inactivating mutations were present in 10 of 93 aGCTs (10.8%), and the frequency of these mutations was similar between primary and recurrent aGCTs. Inactivating mutations, including a splice site mutation in candidate tumor suppressor *WNK2* and nonsense mutations in *PIK3R1* and *NLRC5*, were identified at a low frequency in our cohort. Missense mutations were identified in cell cycle‐related genes *TP53*, *CDKN2D*, and *CDK1*. From these data, we conclude that aGCTs are comparatively a homogeneous group of tumors that arise from a limited set of genetic events and are characterized by the *FOXL2* C402G mutation. Secondary mutations occur in a subset of patients but do not explain the diverse clinical behavior of this disease. As the *FOXL2* C402G mutation remains the main driver of this disease, progress in the development of therapeutics for aGCT would likely come from understanding the functional consequences of the *FOXL2* C402G mutation.

## Introduction

Adult‐type granulosa cell tumors (aGCTs) are the most common type of malignant ovarian sex cord‐stromal tumor (SCST) [1]. They account for 2–5% of all ovarian malignant tumors and 90% of malignant SCSTs [2]. aGCTs occur in perimenopausal women with a median age of diagnosis of between 50 and 54 years [3, 4].These tumors arise from the granulosa cells of the ovarian follicles and have distinct clinical features, such as their ability to secrete estrogen and inhibins [5]. Due to their indolent growth and unique hormonal activity, these tumors are usually diagnosed at an early stage and are treated with surgery [6, 7, 8]. A somatic missense mutation (c.C402G; p.C134W) in the transcription factor *FOXL2* was identified in over 95% of aGCTs and can be used as a clinical diagnostic assay for differential diagnosis [9, 10, 11, 12]. In our previous study by McConechy *et al*, we showed that, in a molecularly defined cohort of aGCTs (*FOXL2* C402G mutation positive), the overall survival did not differ from age‐matched, population‐based controls [13]. However, one‐third of aGCT patients relapse, and there are no effective treatments for relapsed patients with inoperable tumors [14, 15].

Due to the propensity of aGCT to recur years after initial diagnosis (typically 4–8 years, with the median being 7.2 years in molecularly defined cohorts), the challenge is identifying patients who are at high risk for relapsed disease and require prolonged surveillance [13, 14, 16]. As the *FOXL2* C402G mutation is present in essentially all aGCTs, there has been much research on secondary mutations to explain the diverse clinical behavior of these tumors. Our research group was the first to describe *TERT* C228T promoter mutations in 51 of 229 (22%) primary and 24 of 58 (41%) recurrent aGCTs [17]. Likewise, Alexiadis *et al* performed targeted *TERT* promoter sequencing and found that a higher frequency of recurrent tumors harbor the *TERT* C228T promoter mutation [18]. Hillman *et al* were the first to identify *KMT2D* truncating mutations in aGCT and suggested that *KMT2D* inactivation may increase the risk of tumor recurrence [19]. Da Cruz Paula *et al* used targeted sequencing of known cancer genes and identified genetic alterations in cell cycle‐related genes, including *TP53*, *TERT* promoter, *MED12*, and *CDKN2A/B*, suggesting that these mutations may play a role in recurrence [20, 21]. Furthermore, Roze *et al* suggest that patients with *TP53* mutations represent a high‐grade subgroup of aGCT [21].

In this study, we used whole‐genome sequencing (WGS) on 10 aGCTs and their matched peripheral blood to identify novel somatic mutations in aGCT. Validation was performed using targeted sequencing on an independent cohort of 83 primary and recurrent aGCTs collected from four international institutions. We identified *KMT2D* inactivating mutations in 10 of 93 aGCTs (10.8%). A splice site mutation was identified in the candidate tumor suppressor *WNK2*, and nonsense mutations were identified in *NLRC5* and *PIK3R1*. Missense mutations were identified in cell cycle‐related genes *TP53*, *CDKN2D*, and *CDK1*.

## Materials and methods

### Clinical specimens for exploratory WGS

Ten representative fresh‐frozen aGCTs were selected from OVCARE's Gynecological Tissue Bank in Vancouver, Canada, for WGS. Patient tumor and peripheral blood samples were collected at diagnosis during standard‐of‐care debulking surgery. For DNA isolation from banked samples, frozen tissue samples were cryosectioned, and sections adjacent to the scrolls submitted for sequencing were stained with hematoxylin and eosin to assess tumor cell content. These slides were reviewed by two of the following gynecologic pathologists: HMH, ANK, HLC, and BG, and the final cases with >80% tumor cellularity were selected by BG. All cases were positive for the *FOXL2* (c.C402G; p.C134W) mutation. In brief, WGS was performed using Illumina HiSeq 2500 v4 chemistry (Illumina Inc., Hayward, CA, USA) with the polymerase chain reaction (PCR)‐free protocol to eliminate PCR‐induced bias and to improve coverage across the genome. The average read depth was 46.6X per sample (range: 36–60X). WGS analysis comparing tumor tissue to matched peripheral blood was performed to identify somatic alterations in each case, including single‐nucleotide variants (SNVs), small insertions and deletions (indels), copy number alterations (CNAs), and structural variants (SVs). This cohort was previously published by our research team, and detailed methods are available [22].

### Validation cohort description

Formalin‐fixed paraffin‐embedded (FFPE) aGCTs (*n* = 83) were collected from Helsinki University Hospital (Helsinki, Finland; *n* = 39), Massachusetts General Hospital (Boston, MA, USA; *n* = 27), Tübingen University Hospital (Tübingen, Germany; *n* = 13), and Netherlands Cancer Institute (Amsterdam, The Netherlands; *n* = 4). Sections prepared from FFPE tumor blocks were reviewed by one of the following pathologists (BG, FK, HMH, ANK, RB, HLC, BT‐C, and LH) to confirm aGCT diagnosis. DNA was extracted for each FFPE tumor block, and the *FOXL2* C402G mutation was confirmed with a single‐nucleotide polymorphism (SNP) genotyping assay using two allele‐specific fluorescent labelled probes. The respective institutional research ethics board approved the waiver for patient consent. The University of British Columbia and BC Cancer's Research Ethics Boards approved the overall project methods.

### Targeted sequencing

To explore the frequency of variants identified in the WGS study of 10 aGCTs, a custom amplicon‐based panel was designed to sequence a larger validation cohort of FFPE cases. The genes included in the custom panel were prioritized by (1) genes with frameshift (small insertions/deletions) or nonsense or splice site mutations, (2) genes with SNVs in known cancer genes, and (3) genes with SNVs involved in granulosa cell biology. The full list of genes is given in supplementary material, Table S1. Two sets of custom oligo capture probes flanking each gene of interest were designed for the construction of two different sequencing libraries (A and B). These custom probes amplified the entire coding region of 39 genes selected in the custom panel. TruSeq Custom Amplicon Dual Strand Library Preparation was performed to generate 1536 amplicons (150 base pairs each) per sample in a single multiplex reaction using the manufacturer's protocol (Illumina Inc.). PCR was used to incorporate both a unique index for each sample and sequencing primers. For each sequencing run, 24 samples were pooled into a single library with a final concentration of 4 nm. The pooled library was sequenced on an Illumina MiSeq using the MiSeq Reagent Kit V2. The average read depth was 1254X per sample (range: 296–3212X).

### Case inclusion

**Figure 1:**
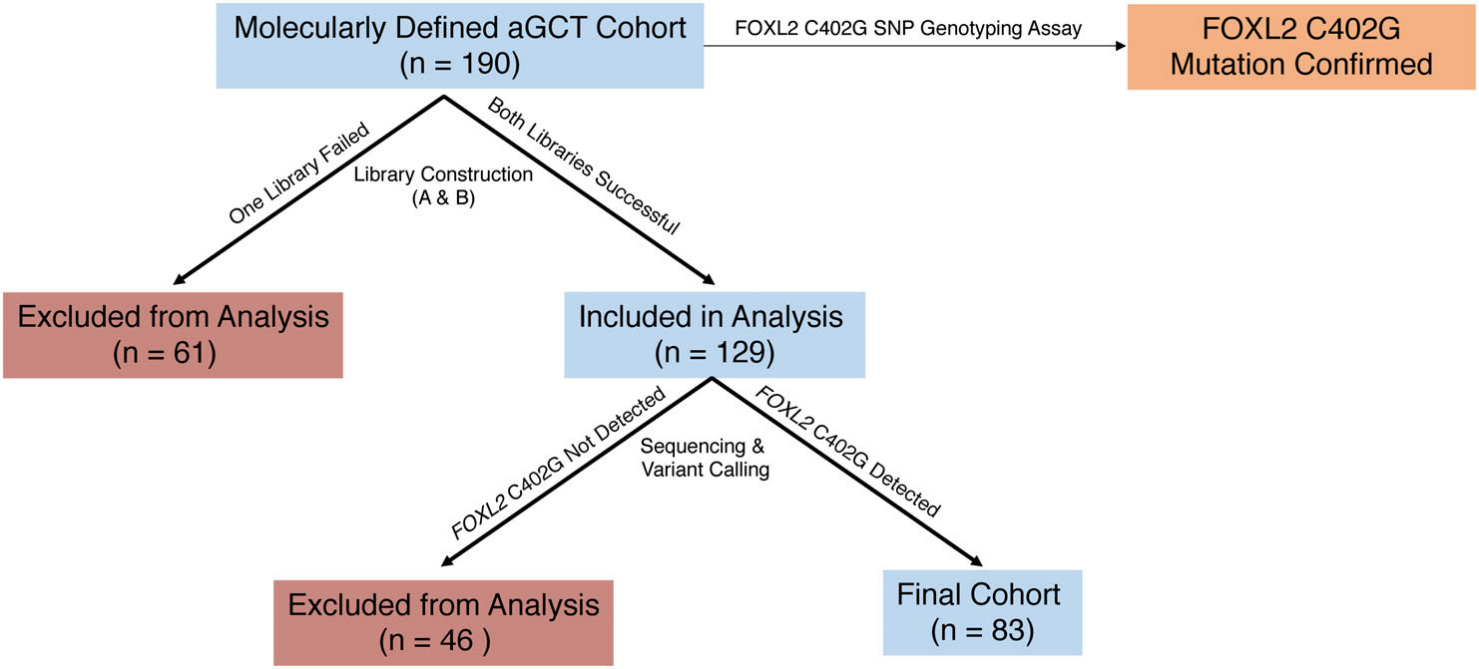
Validation cohort selection and case inclusion. FOXL2 C402G mutation was confirmed in all available cases (*n* = 190) using an SNP genotyping assay. Two libraries were constructed for each case. Cases where one library construction failed were excluded (*n* = 61). Cases where both libraries were successful were sequenced, and variants were called (*n* = 129). Cases where the previously confirmed FOXL2 C402G mutation was not detected using the targeted sequencing variant analysis pipeline were excluded (*n* = 46). The final cohort consisted of cases with two libraries and where FOXL2 C402G mutation was detected using the targeted sequencing variant analysis pipeline (*n* = 83).

All cases were tested for *FOXL2* C402G mutation using an SNP genotyping assay and were confirmed to contain the *FOXL2* C402G mutation. As the validation cohort consisted of FFPE material with no matched normal tissue available, the cases included in the final cohort reported were selected if they satisfied the following two conditions: (1) two libraries (A and B) per case were constructed successfully and sequenced, and (2) *FOXL2* C402G mutation was detected using the targeted sequencing variant analysis pipeline to call mutations in the gene panel. Condition 1: Two libraries (A and B) per case are important to eliminate any sequencing artifacts that may have resulted from the formalin fixation process. Libraries A and B were constructed with different probes to generate amplicons covering the 39 genes selected in our custom gene panel. Both libraries A and B were sequenced separately and processed independently through the variant analysis pipeline. After the variants were called for both libraries A and B, an intersection function was performed that generated the list of variants that were present in both libraries A and B. The assumption that formalin fixation introduces SNVs at random enabled the removal of these randomly generated SNVs that were only present in one of the two libraries. The mutations reported in the Results section were present in both libraries A and B for each case. Condition 2: The detection of the *FOXL2* C402G mutation using the targeted sequencing variant analysis pipeline was employed to ensure that the targeted sequencing pipeline was highly sensitive. The aim of the study was to report on a molecularly defined aGCT cohort, where all cases harbor the *FOXL2* C402G mutation. Therefore, cases were tested beforehand using an SNP genotyping assay to confirm the presence of the *FOXL2* C402G mutation, and only cases where the *FOXL2* C402G mutation was called using the targeted sequencing variant analysis pipeline were included in the validation cohort. This ensured that the pipeline was highly sensitive in detecting variants in the remaining genes in the panel. Due to the stringency required to remove FFPE artifacts and the fact that some of the cases are over 50 years old with no matched normal tissue available, it was expected that there would be a high number of cases excluded from the validation cohort. A less rigorous approach would lead to the inclusion of an overwhelming number of false‐positive mutations. A flowchart outlining the number of cases excluded at each step is illustrated in Figure 1.

### Variant calling and filtering

SNVs were detected by MutationSeq version 4.3.7 (Vancouver, Canada) and Strelka version 1.0.14 (Illumina Inc., San Diego, CA, USA). Small insertions and deletions (indels) were detected using Strelka version 1.0.14 and Mutect2 version 4.1.8.1 from GATK (The Broad Institute, Cambridge, MA, USA). SNVs were removed if they were not detected in both libraries A and B and by both MutationSeq and Strelka. Indels were removed if they were not detected in both libraries A and B and by both Mutect2 and Strelka. Variants with a read depth <100 and SNVs from the Single Nucleotide Polymorphism Database (dbSNP) were removed. The presence of the *FOXL2* C402G mutation in all the cases included in validation cohort was confirmed prior to targeted sequencing. The lowest variant allele frequency (VAF) in which *FOXL2* C402G was detected was 8.5% using the variant analysis pipeline on the targeted sequencing data. This established the limit of the detection for identifying the *FOXL2* C402G mutation and was thus chosen as the VAF for variant reporting of the remaining genes in the panel. Furthermore, the *FOXL2* C402G mutation is expected to be clonal with secondary subclonal mutations. For missense mutations, three tools (PolyPhen‐2, SIFT, and MutationAssessor) were selected to predict the pathogenicity of each variant using Ensembl's Variant Effect Predictor. Variants that were predicted by two or more tools to have no effect on protein function were removed. Therefore, only missense mutations that were predicted as pathogenic by two or more tools were reported. A flowchart describing the steps of variant filtering is illustrated in supplementary material, Figure S1.

## Results

### WGS on exploratory cohort

Ten aGCTs (eight primary and two recurrent) and their matched normal DNA samples were subjected to WGS as previously described [22]. The median age of diagnosis was 51 years for aGCT, and the clinical characteristics are described in Table 1. All aGCTs were heterozygous for the *FOXL2* C402G mutation. Somatic variations were detected in the tumor genome of each patient, including SNVs, small indels, CNAs, and SVs. The overall mutational burden for these 10 aGCTs was low as previously described in the Wang *et al*'s study [22]. SNVs and small indels are described in Table 1. The majority of mutations reported was identified in only 1 of 10 aGCTs (10%) except for the *TERT* C228T promoter mutation that was present in 5 of 10 aGCTs (50%). Missense mutations in *BMP7* and *MPHOSPH8* were identified in 2 of 10 aGCTs (20%). Copy number analysis revealed loss of chromosome 22 in 6 of 10 aGCTs (60%) and gain of chromosome 14 in 2 of 10 aGCTs (20%). Concurrent loss of chromosome 22 and gain of chromosome 14 was observed in 1 of 10 aGCTs (10%). No gain of chromosome 12 was found. These results confirm previous reports of CNAs in aGCTs, including loss of chromosome 22 in 30–56% and gain of chromosome 14 in 20–27% [18, 21, 23, 24, 25]. Other structural rearrangements were sporadic and infrequent (see supplementary material, Figure S2).

**Table 1:** Clinical features of patients and mutations in the exploratory cohort.

Patient	Age	Diagnosis	Stage	Disease	Menopause status	Tumor size	Adjuvant treatment	Mutations (protein change)
DG1331	63	aGCT	Unk	Recurrent	Unk	Unk	No	DOCK3 S1702F, TSKU W237*, DNMBP R340fs
DG1332	76	aGCT	Unk	Recurrent	Post	7 × 4 × 4 cm	Palliative radiation	CDKN2D V24G, KDR R299W, NLRC5 R1161K, PIK3CA G1007R, SLITRK2 R713*, WNK2 L1004F, ACSS1 I134fs, GANC Y249fs, *TERT* C228T promoter^†^
DG1333	73	aGCT	I	Primary	Post	Unk	Chemotherapy	CENPE L2378W, KDM5C W555R, MPHOSPH8 L590F, TP53 K121N, KMT2D S1398fs
DG1334	61	aGCT	IA	Primary	Peri	5.5 × 3.5 × 2 cm	No	CCR5 G21C
DG1335	59	aGCT	I	Primary	Pre	Unk	No	KCMF1 Y28*, MPHOSPH8 L590F, NDUFA10 K285N, NDUFA10 Q286*, FOXL2 A243fs, POLR1B P1084fs
DG1336	60	aGCT	IC	Primary	Post	15 × 8 × 8 cm	No	BMP7 R188W, NOX1 F185L, GRIA3 W137fs, *TERT* C228T promoter^†^
DG1337	64	aGCT	Unk	Primary	Unk	10 × 7.5 × 3.5 cm	Unk	KANSL3 Q372*, SMAD3 L135P, ZP1 R552fs, *TERT* C228T promoter^†^
DG1338	37	aGCT	I	Primary	Pre	Unk	No	
DG1339	43	aGCT	IA	Primary	Pre	24 × 11 × 9 cm	No	GLI1 G544E, PIK3R1 M582_splice, WDR72 D201fs, *TERT* C228T promoter^†^
DG1340	66	aGCT	IC	Primary	Post	17 × 13 × 6 cm	No	BMP7 N289K, CDK1 M223V, WDR52 R1655*, TRO I543fs, *TERT* C228T promoter^†^

fs, frameshift mutation; splice, splice site mutation; Unk, unknown.

* Stop codon, nonsense mutation.

^†^ DNA sequence change.

### Targeted sequencing on validation cohort

**Figure 2:**
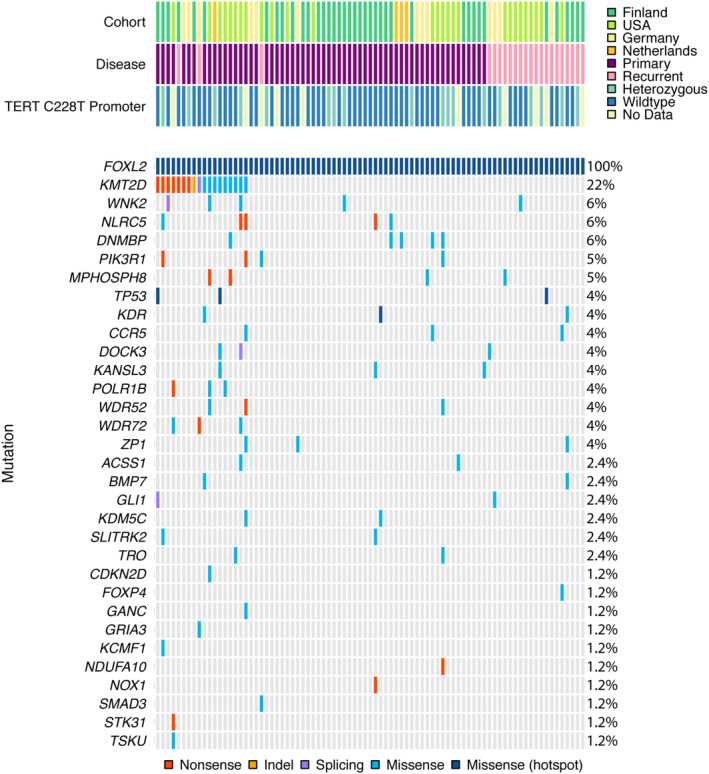
Mutations in aGCTs. OncoPrint showing the distribution of genetic alterations in aGCTs. Genetic alterations in genes (rows) on targeted sequencing panel are shown in each aGCT case (columns). The nonsense, indels (insertions/deletions), splice site, and missense mutations are shown in red, yellow, purple, and blue, respectively. Missense mutations that are putative driver mutations (hot‐spot mutations) are colored in dark blue. The percentage of cases harboring mutations in each gene is annotated on the far right. The country from which each case was collected from, the disease status (primary or recurrent), and *TERT* C228T promoter mutation status are annotated on the tracks above the OncoPrint.

Our validation cohort encompassed 83 aGCTs, including 61 primary and 22 recurrent tumors, collected from four international institutions. For the 58 patients with age data, the median age at diagnosis was 52 years (range: 26–94 years). The clinical features for each cohort are described in Table 2. All cases included in the validation cohort have confirmed *FOXL2* C402G mutation determined by an SNP genotyping assay, and this mutation was detected in the targeted sequencing for all cases reported (Figure 1). Two cases harbor *FOXL2* C402G hemizygous mutations, which appear as a homozygous state. The remaining cases were *FOXL2* C402G heterozygous. For the 73 aGCTs with available *TERT* C228T promoter mutation data, the frequency was 18 of 73 aGCTs (24.7%), which is similar to our original *TERT* C228T promoter mutation publication that contains additional cases along with 73 cases of the current validation cohort (75/287; 26.1%). The following missense mutations were mutated in two aGCTs: *KMT2D* (c.C3893T; p.S1298F) and *NLRC5* (c.G823A; p.E275K). All genes mutated in our panel are shown in Figure 2 and in supplementary material, Table S2. *KMT2D* inactivating mutations (nonsense, indel, or splice site mutations) were present in 9 of 83 aGCTs in our validation cohort, and an indel (frameshift mutation) was reported in 1 case from the exploratory cohort (10/93; 10.8%). Missense mutations in *KMT2D* were observed in 9 of 93 aGCTs (9.7%); however, these mutations have not previously been reported, and therefore, their relevance to aGCT development is unknown. The missense mutations annotated in dark blue in Figure 2 are putative driver events and include mutations in *FOXL2*, *TP53*, and *KDR*. The significance of the remaining missense mutations annotated in light blue remains unknown. Splice site mutations were identified in *KMT2D*, *WNK2*, *DOCK3*, and *GLI1*. Nonsense mutations were identified in *KMT2D*, *NLRC5*, *PIK3R1*, *POLR1B*, *WDR52*, *WDR72*, *NDUFA10*, *NOX1*, and *STK31*. Moreover, genes involved in cell cycle regulation were mutated at a low frequency in aGCT, including *TP53* (4/93; 4.8%), *CDKN2D* (2/93; 2.1%), and *CDK1* (1/93; 1.2%). Mutual exclusivity of *TP53* and *TERT* C228T promoter mutations was observed for the three cases with available *TERT* C228T promoter mutation data.

**Table 2:** Clinical features of patients in the validation cohort.

Cohort	Finland	USA	Germany	The Netherlands
Number of patients	39	27	13	4
Median age	52	Unknown	46	53.5
Stage				
I	36	0	8	3
II	2	0	1	0
III–IV	1	0	1	1
Unknown	0	27	3	0
Disease				
Primary	32	17	8	4
Recurrent	7	10	5	0
Adjuvant treatment				
No	34	0	9	4
Chemotherapy	4	0	1	0
Radiation	1	0	0	0
Unknown	0	27	3	0
FOXL2 C402G mutation status				
Homozygous/hemizygous	1	1	0	0
Heterozygous	38	26	13	4
*TERT* C228T promoter mutation				
Heterozygous	8	7	2	1
Wildtype	27	13	11	3
Unknown	4	6	0	0

## Discussion

Here, we report the genomic analysis of 93 aGCTs, a malignant SCST of the ovary associated with late recurrence. The current study is a targeted genomic analysis of a molecularly defined aGCT cohort, where all tumors harbor the *FOXL2* C402G mutation. Our research team initially described *TERT* C228T promoter mutations in 51 of 229 primary (22%) and 24 of 58 recurrent (41%) aGCTs, suggesting that these mutations occur later in tumorigenesis. The majority of the previously described cohort is included in this study, and *TERT* C228T promoter mutations are annotated in Figure 2 and Table 2. Multiple research groups have also observed *TERT* promoter mutations with a higher frequency of mutations in recurrent aGCTs (6/9, 67%; 1/28, 64%) compared to primary aGCTs (5/17, 29%; 5/19, 26%) [18, 20]. In our study and that of Alexiadis *et al*, seven and five patients whose tumors were initially wild‐type for the *TERT* promoter acquired the C228T mutation in their recurrent tumors, respectively [17, 18]. These data support our statement that *TERT* promoter mutations are acquired during tumor progression after the initial *FOXL2* C402G driver mutation. Although targeting telomerase as an anticancer treatment may represent an attractive strategy for recurrent aGCT, the development of efficacious cancer‐specific telomerase inhibitors remains an ongoing challenge [26].

Using a combination of WGS and targeted sequencing, the results of the current study demonstrate that *KMT2D* undergoes frequent inactivating mutations in aGCTs. We report a similar frequency of *KMT2D* inactivating mutations (10/93, 10.8%) as the Hillman *et al*'s study, which reported that 11 of 79 (13.9%) aGCTs harbored *KMT2D* inactivating mutations. Hillman *et al* described inactivating mutations in *KMT2D* as a recurrent event (10/43, 23%) in these tumors compared to primary tumors (1/35, 3%) [19]. However, in our dataset, we observed a similar frequency of inactivating mutations in *KMT2D* in primary (8/69, 11.5%) and recurrent (2/24, 8.3%) aGCTs. Aligned with our current results, Da Cruz Paula *et al* identified only one inactivating *KMT2D* mutation in a primary nonrecurrent aGCT in a cohort consisting of 10 primary nonrecurrent aGCTs (defined by no recurrent tumor within 4 years of initial diagnosis), 28 recurrent aGCTs, and 9 matched primary aGCTs with known recurrences [20]. The occurrence of *KMT2D* inactivating mutations in 10.8% of our aGCT cohort not only suggests that they are a pathogenic driver mutation in a subset of aGCTs but also that these mutations do not correlate with recurrence. We also observed *KMT2D* missense mutations in a subset of aGCTs (9/93; 9.7%), but these variants are of unknown significance. A limitation of this study was that the validation cohort did not have matched normal tissue, and thus, some reported missense mutations may be rare SNPs. Further research into the role of *KMT2D* in aGCT development is warranted, specifically exploring the epigenetic landscape of these tumors to identify aberrant histone marks and methylation patterns.

Additional inactivating mutations, including a splice site mutation in the candidate tumor suppressor *WNK2* and nonsense mutations in the newly discovered protein NLRC5, are reported. *WNK2* was recently discovered to be epigenetically silenced through promoter hypermethylation in gliomas and meningioma, suggesting its role as a growth suppressor [27, 28, 29]. *WNK2* was also found to be downregulated via epigenetic silencing in early pancreatic ductal adenocarcinoma and may support cell proliferation through the Mitogen‐activated protein kinase (MAPK) signaling pathway [30]. *WNK2* somatic mutations and copy number loss were reported in hepatocellular carcinoma, resulting in lower WNK2 protein levels, which were associated with early tumor recurrence linked to enhanced ERK1/2 signaling [31]. Moreover, WNK kinases have been linked to Wnt/β‐catenin signaling, a known pathway involved in granulosa cell development, and loss‐of‐function *WNK* genes resemble canonical Wnt pathway mutants [32]. NLRC5 has recently been linked to the regulation of cancer immune evasion, but its role in tumor development is controversial [33]. Some research studies report that NLRC5 elicits antitumor immunity, while other studies found that it promoted tumorigenesis and progression [33]. In fact, NLRC5 has been shown to regulate the Wnt/β‐catenin signaling pathway to promote cell proliferation and migration in both clear cell renal carcinoma and hepatocellular carcinoma [34, 35].

Various signaling pathways have been explored in the development of aGCT, including transforming growth factor beta (TGF‐β), phosphatidylinositol‐3‐kinase; serine/threonine kinase (PI3K/AKT), GATA4, and vascular endothelial growth factor (VEGF). These factors have been shown to play important roles in granulosa cell proliferation, apoptosis, or angiogenesis. In our aGCT cohort, we observed a small number of mutations in members of these pathways including *GL1*, *BMP7*, *SMAD3* (TGF‐β pathway), *PIK3R1* (PI3K/AKT pathway), *GATA4*, and *KDR/VEGFR2*. However, these mutations occurred in a small percentage of aGCT patients and do not provide any prognostic or predictive value. In addition, mutations in cell cycle‐related genes, including *TP53*, *CDKN2D*, and *CDK1*, were identified.

Da Cruz Paula *et al* used targeted sequencing of over 400 cancer‐related genes on a cohort of 38 aGCT patients. They identified mutations present in recurrent aGCTs that were not present in primary aGCT, including *TERT* promoter mutations (C228T and C250T), *MED12*, and *TP53*, as well as *CDKN2A/B* homozygous deletions, suggesting that genetic alterations in cell cycle‐related genes may be associated with recurrence [20]. In the current cohort, *TP53* mutations were observed in three primary and one recurrent aGCTs, *CDKN2D* mutations were observed in one primary and one recurrent aGCT, and a *CDK1* mutation was observed in one primary aGCT. The frequency of mutations in cell cycle‐related genes is low in our cohort; thus, it is difficult to draw conclusions regarding their association with recurrence.

Most recently, Roze *et al* used WGS of 33 aGCT patients and identified *TP53* mutations in three patients (3/33; 9.1%) with higher tumor mutational burden and mitotic activity. They propose that tumors with *TP53* mutations define a high‐grade subgroup of aGCT and suggest that a personalized medicine approach is required for treatment of aGCTs. Furthermore, they state that the absence of the *FOXL2* C402G mutation does not exclude an aGCT diagnosis and that *TP53*, *TERT* C228T promoter, and *DICER1* mutations may drive tumorigenesis [21]. The data presented here also show that aGCT can occasionally harbor other driver mutations, including *KMT2D* (10/93; 10.8%) and *TP53* (4/93; 4.8%). However, only a small minority of patients would benefit from a genomic‐driven approach to treatment. The *FOXL2* C402G mutation is the dominant genetic event, and greater benefits would be derived from targeting the downstream mutations.

Inclusively, this study and other research studies have not identified any common actionable features in aGCT. Although it is still possible that features such as promoter methylation could be discovered in noncoding regions, it appears that aGCT is not a disease that could be stratified into different treatment groups by genomic features. Our data and those of others suggest that this is a highly specific clinical entity driven by the *FOXL2* C402G mutation, and treatment strategies focusing on targeting FOXL2 or the downstream consequences are more likely to be successful in treatment compared to a precision medicine approach. Two recent publications have shown initial evidence that the *FOXL2* C402G mutation alters DNA‐binding specificity of the FOXL2 C134W protein (FOXL2^C134W^). Carles *et al* identified unique targets of FOXL2^C134W^, including SLC35F2, a solute transporter that transports sepantronium bromide, which is a transcriptional suppressor of survivin. They found that the immortalized granulosa cell line SVOG3e transduced with inducible FOXL2^C134W^ showed an increased sensitivity to YM155, a small molecule inhibitor of survivin, compared to FOXL2^wild‐type^ or empty vector. The authors suggest YM155 as a potential therapeutic strategy for aGCT [36]. Weis‐Banke *et al* show that the mutant FOXL2^C134W^ acquires the ability to bind SMAD4, which forms a FOXL2^C134W^/SMAD4/SMAD2/3 complex that is able to bind to a novel hybrid DNA motif and induces the transcription of genes involved in epithelial‐to‐mesenchymal transition (EMT). They show that the ablation of SMAD4 or SMAD2/3 in the immortalized granulosa cell line HGrC1 reduces binding of FOXL2^C134W^ to these hybrid sites and decreases expression of these EMT‐related genes, suggesting the possibility of targeting this FOXL2^C134W^–SMAD4 interaction for therapeutic purposes [37]. These studies may lead to the first generally applicable, biologically informed therapeutic strategies for aGCT of the ovary.

## Author contributions statement

JAP, DRC and DGH conceived and designed the study. AEMF, HMH, HSvM, AW, EO, JK, SK, FK, BG and DGH provided study materials or patients. JAP, DRC, SJN and JS collected and assembled data. JAP, DRC, YKW, DL, AB, BHM and ADM analyzed and interpreted data. JAP, DRC and DGH wrote the manuscript. All authors approved the final manuscript.

## Supporting information


**Figure S1.** Variant calling and filtering of targeted sequencing data
**Figure S2.** Circos plots of aGCTs from the WGS exploratory cohort

**Table S1.** Design and selection of genes for custom panel sequencing

**Table S2.** Mutations in the aGCT validation cohort
