# Supplementary material for: Adult‐type granulosa cell tumor of the ovary: a FOXL2‐centric disease
Source: J Pathol Clin Res. 2021 Jan 11;7(3):243–52. doi: 10.1002/cjp2.198 (PMC8072996; doi:10.1002/cjp2.198)
Supplement: Supplementary file 1 — Figure S1. Variant calling and filtering of targeted sequencing data Figure S2. Circos plots of aGCTs from the WGS exploratory cohort [file CJP2-7-243-s003.docx]

**Adult-type granulosa cell tumor of the ovary: a *FOXL2*-centric disease**

JA Pilsworth *et al*. *J Pathol Clin Res* DOI: 10.1002/cjp2.198

**Supplementary Figures S1 and S2**

**
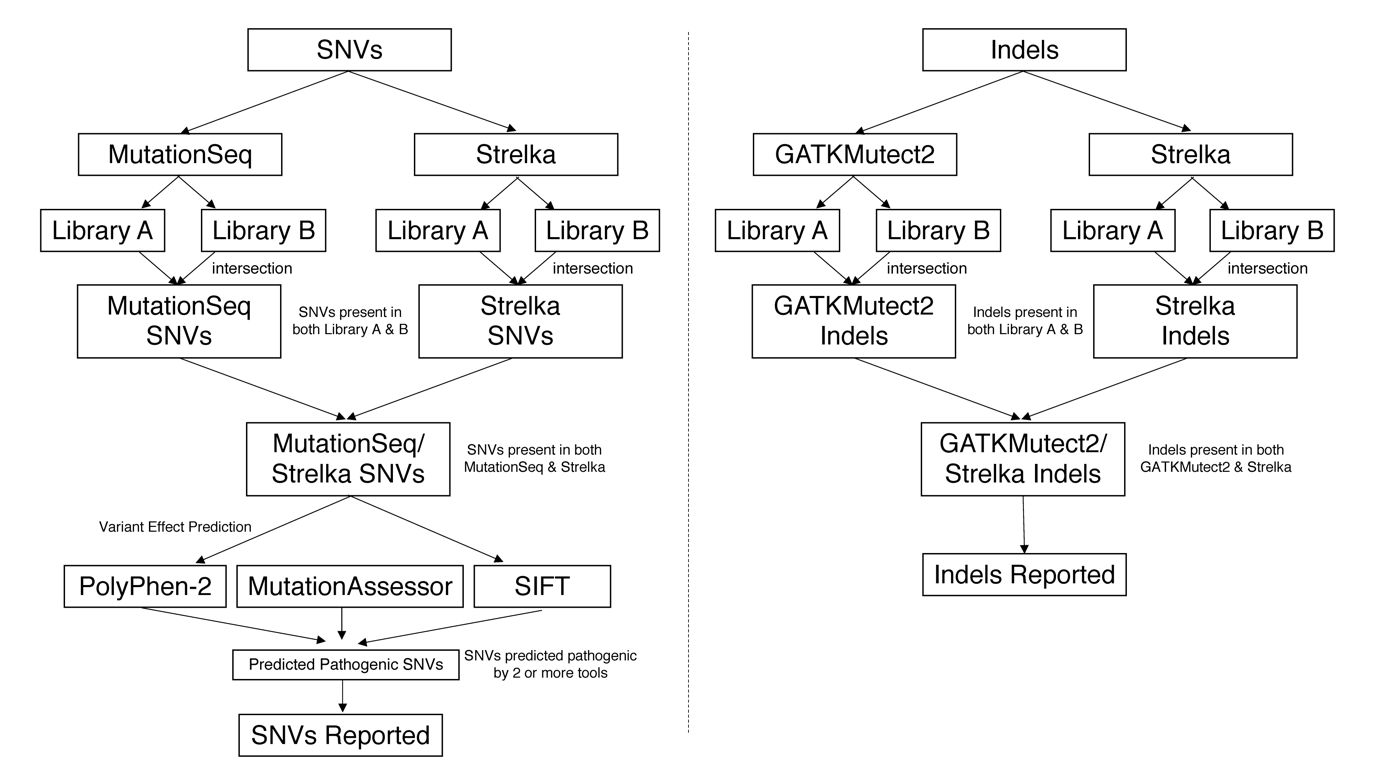
**

**Figure S1.**  Variant calling and filtering of targeted sequencing data. A two-step filtering process was employed to call variants for both SNVs and indels. First, variants were called in both library A and B using each variant caller. Variants that were present in both libraries were kept and the remaining variants were removed. SNVs called by both MutationSeq and Strelka were kept and reported. For missense variants, three variant effect prediction tools (PolyPhen-2, MutationAssesor and SIFT) were employed to remove variants that were predicted to have no effect on protein function (benign). Variants that were predicted to be pathogenic by two or more tools were reported.  A similar approach to indels were used except the tools employed were Mutect2 from GATK and Strelka. Abbreviations: SNVs – single nucleotide variants; indels – insertions/deletions


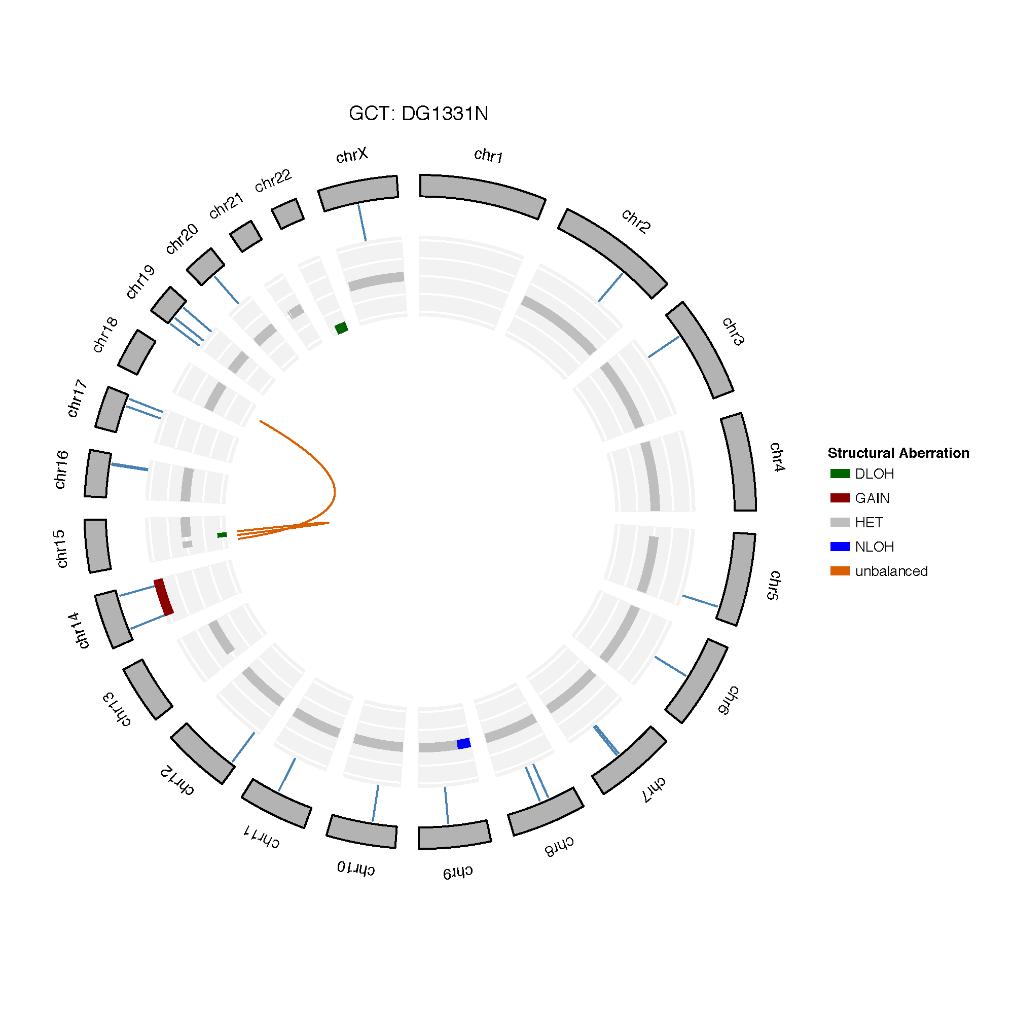


**
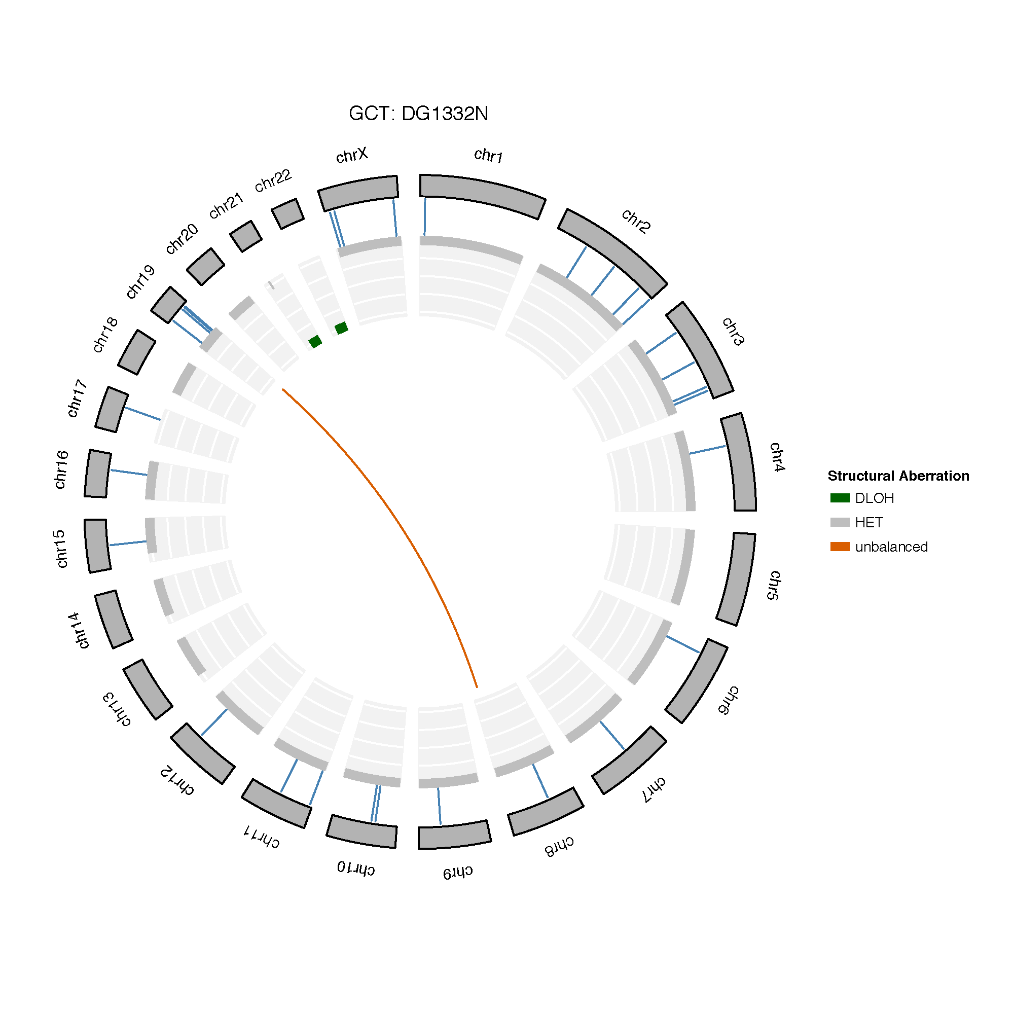
**

**
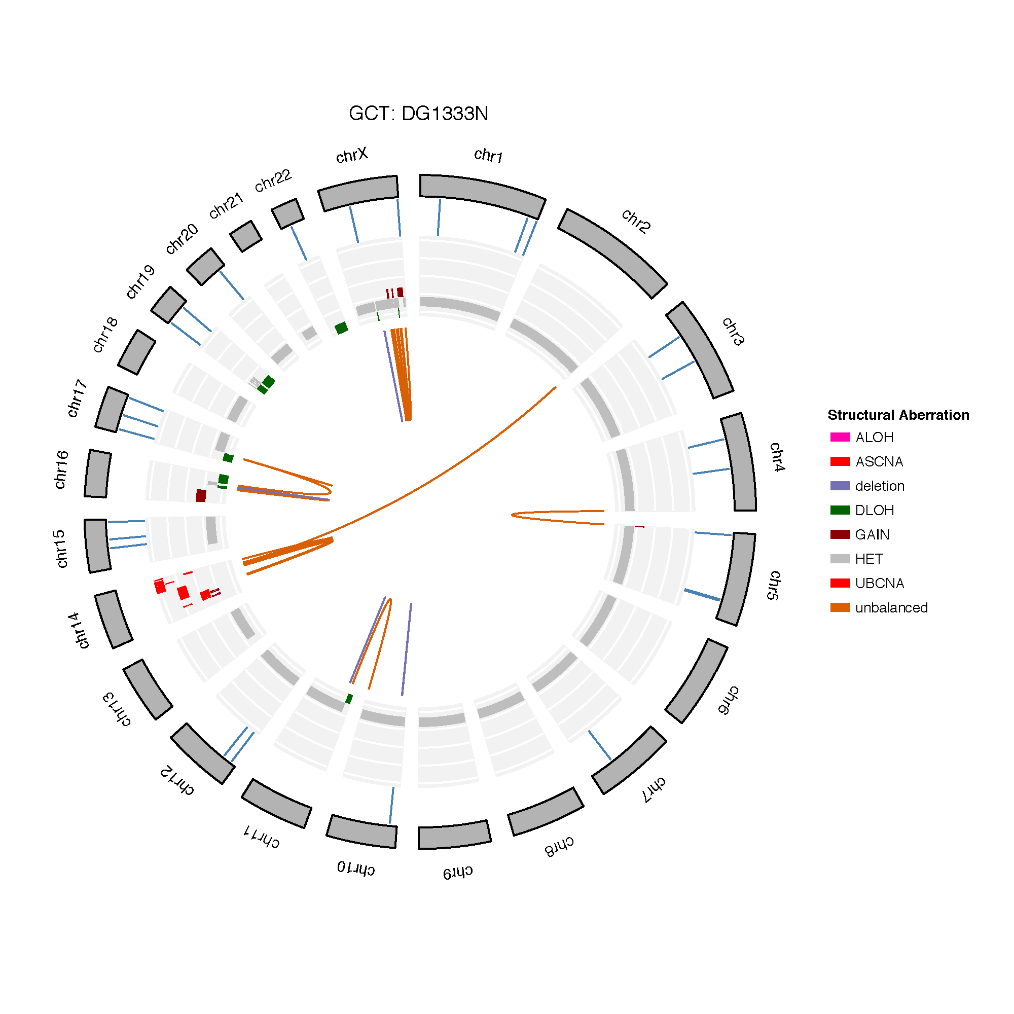
**

**
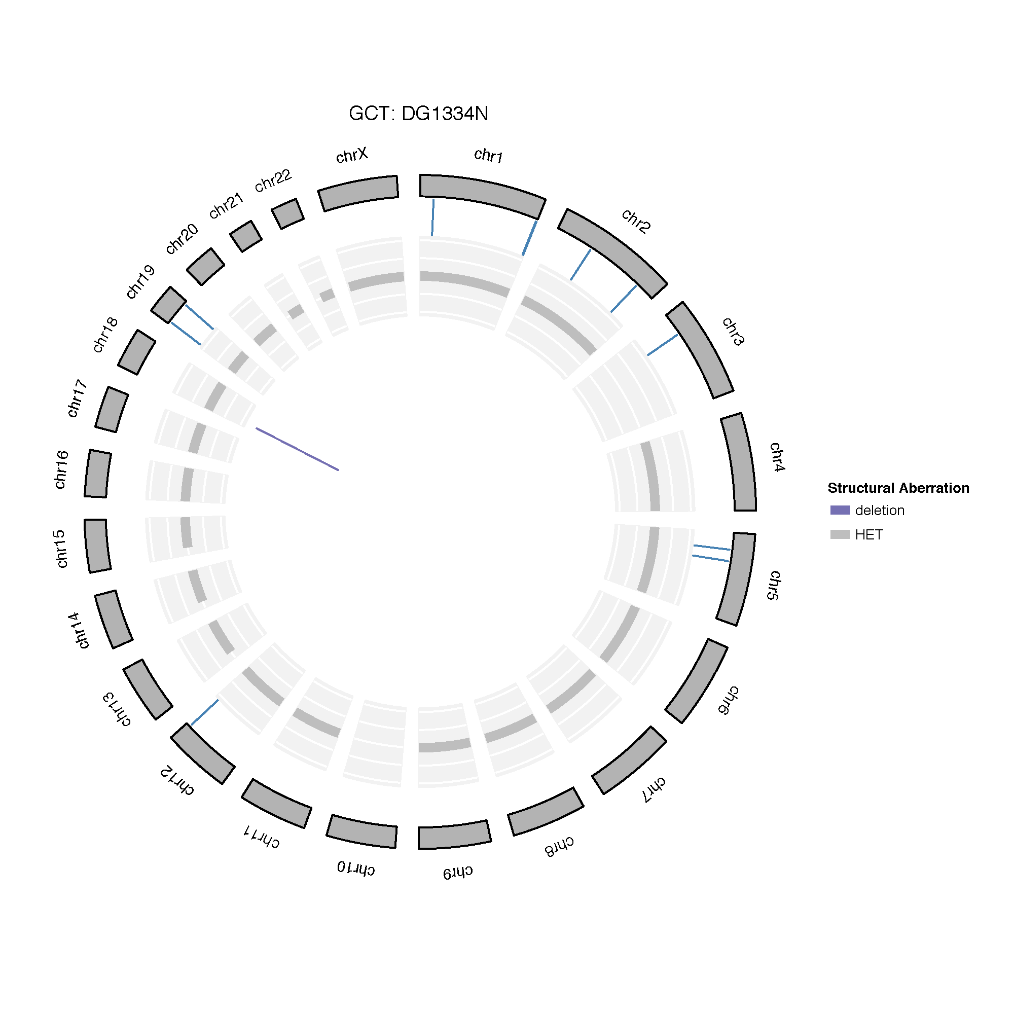
**

**
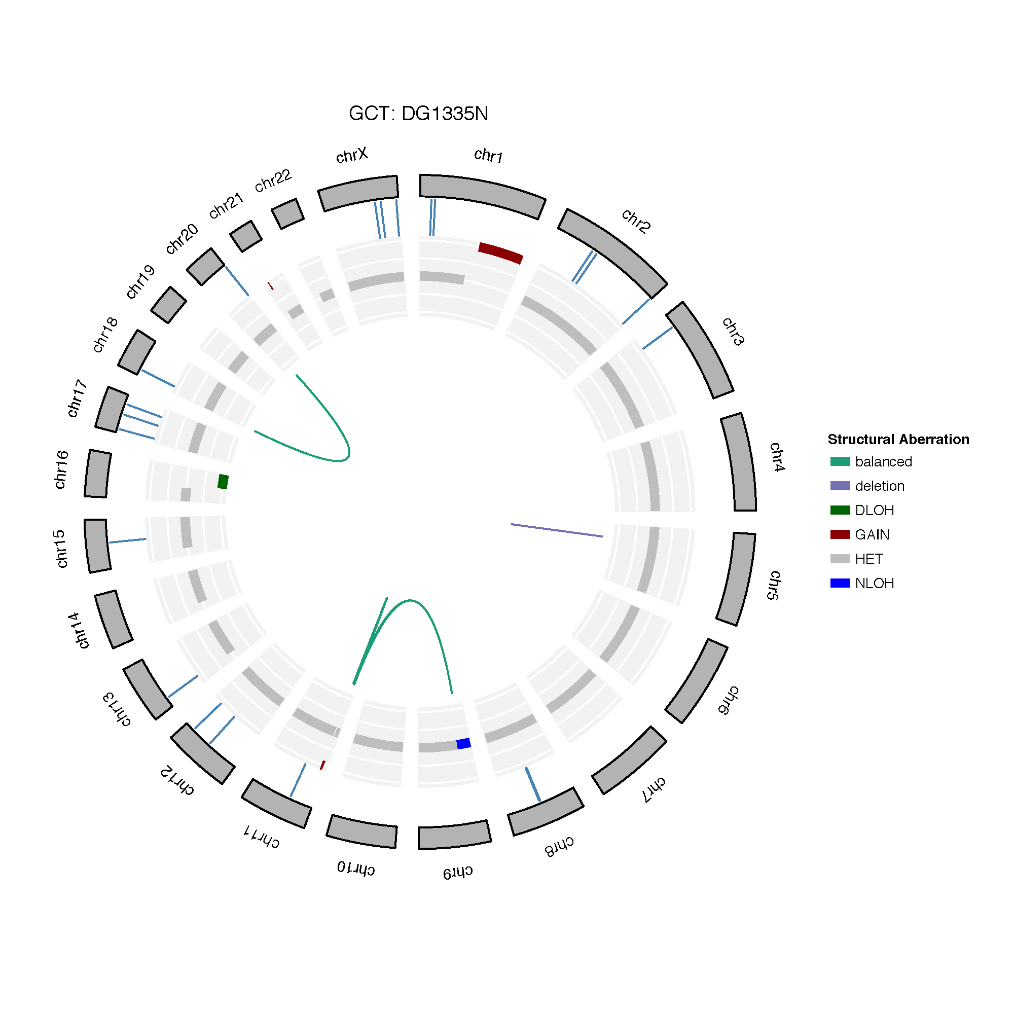
**

**
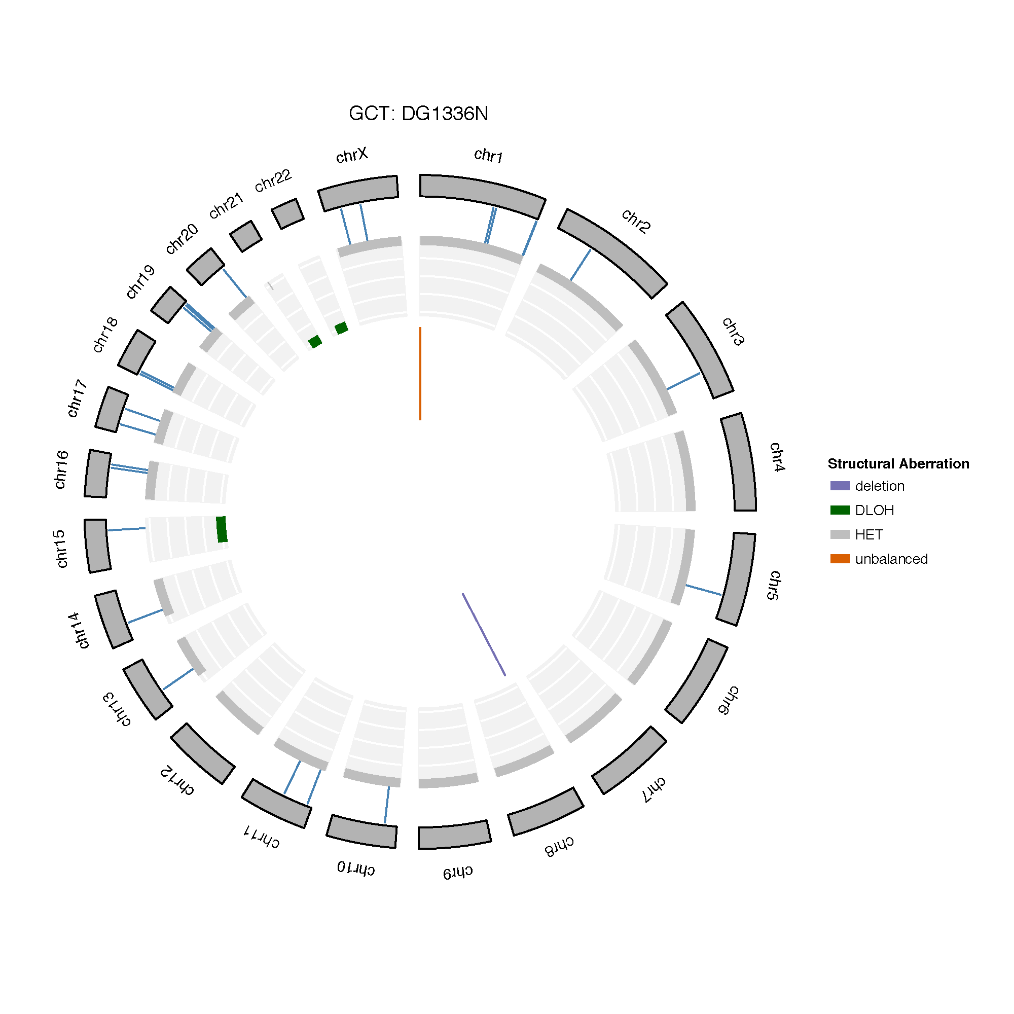
**

**
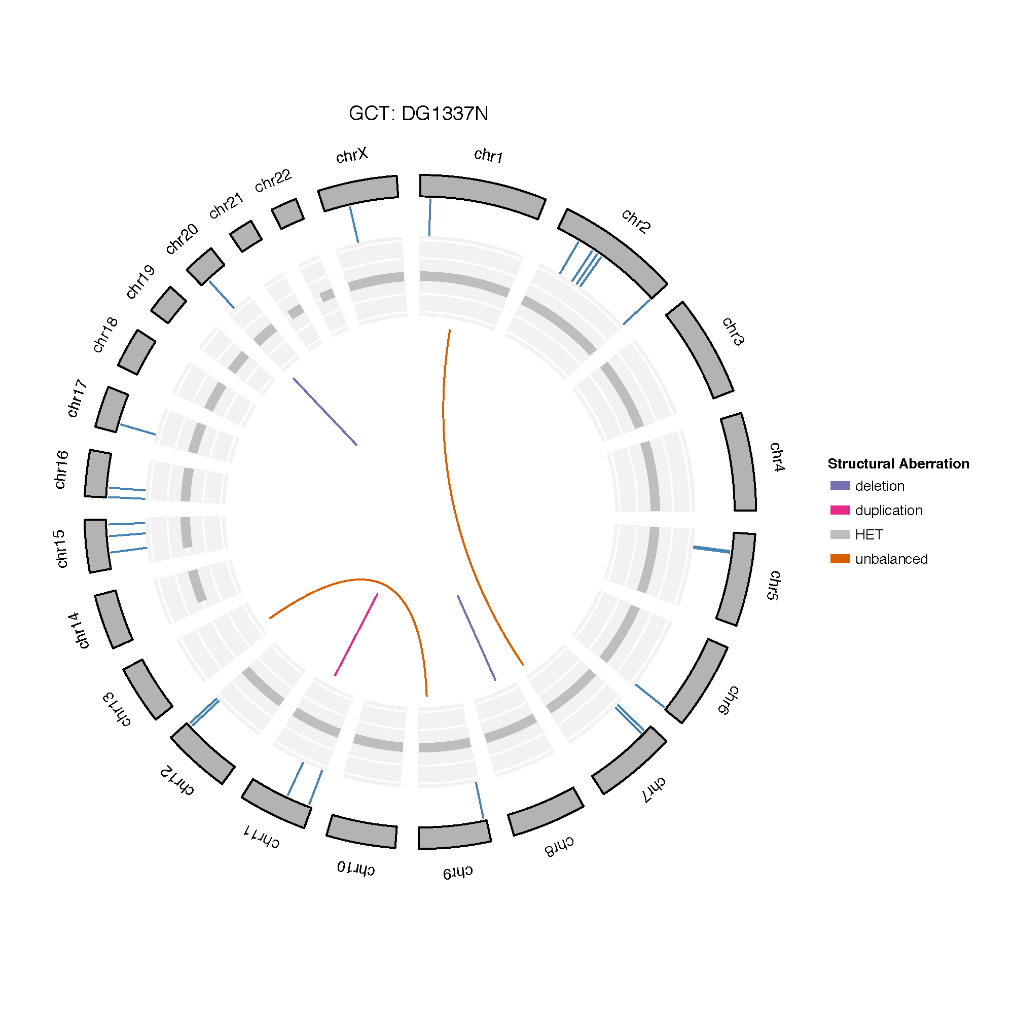
**

**
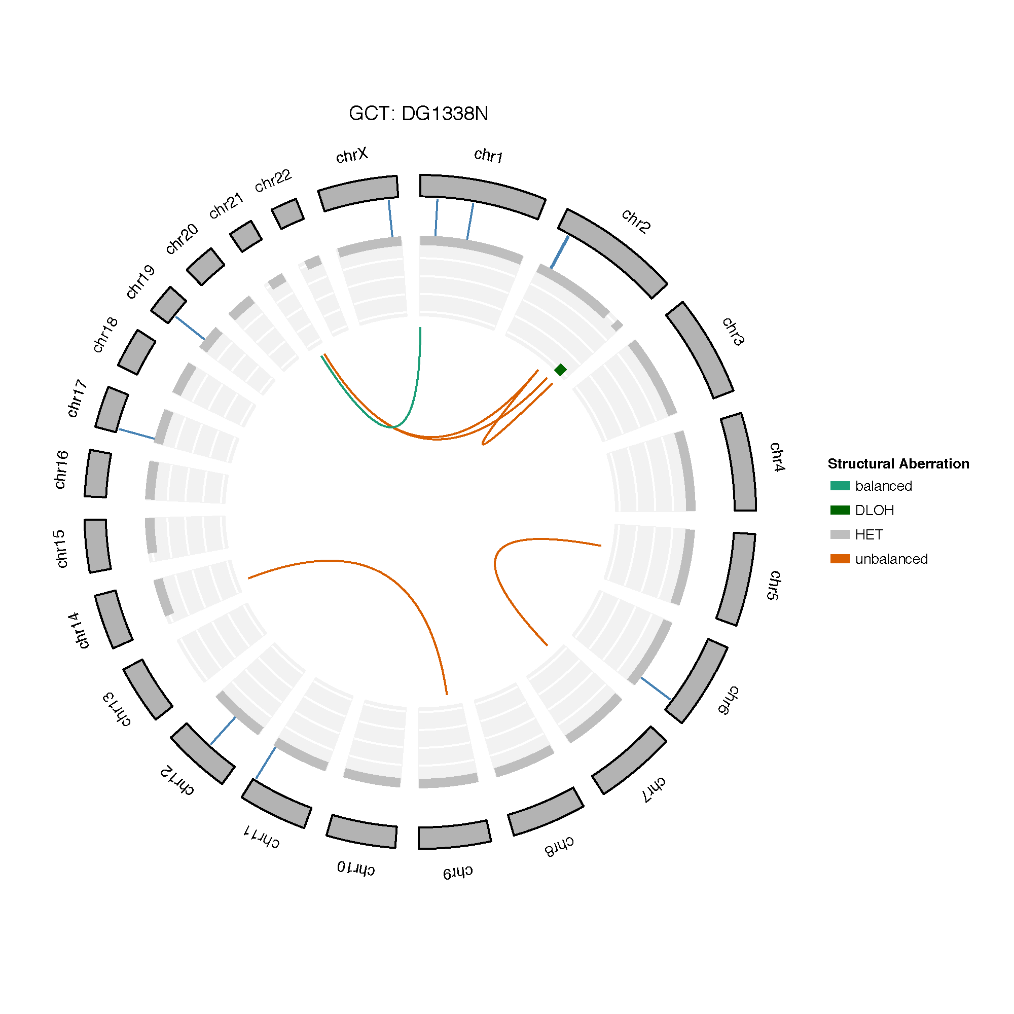
**

**
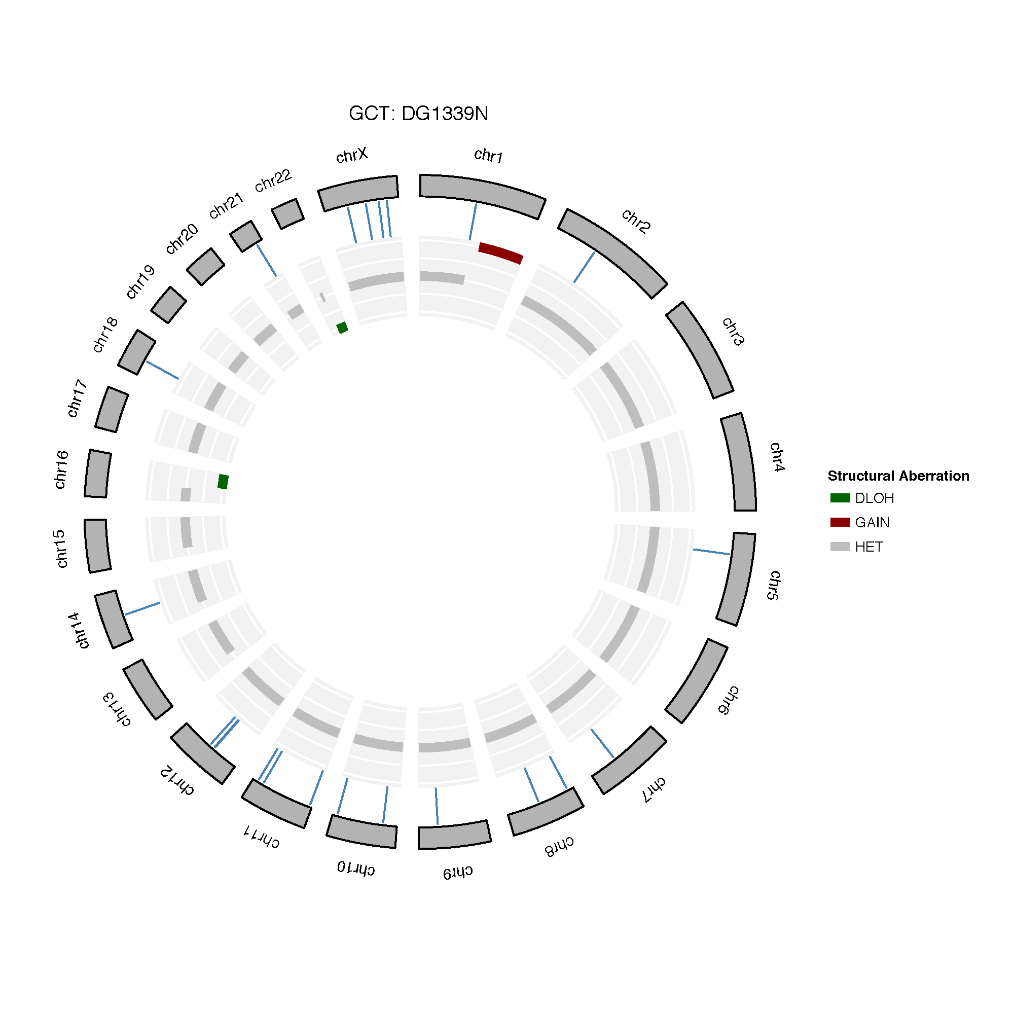
**

**
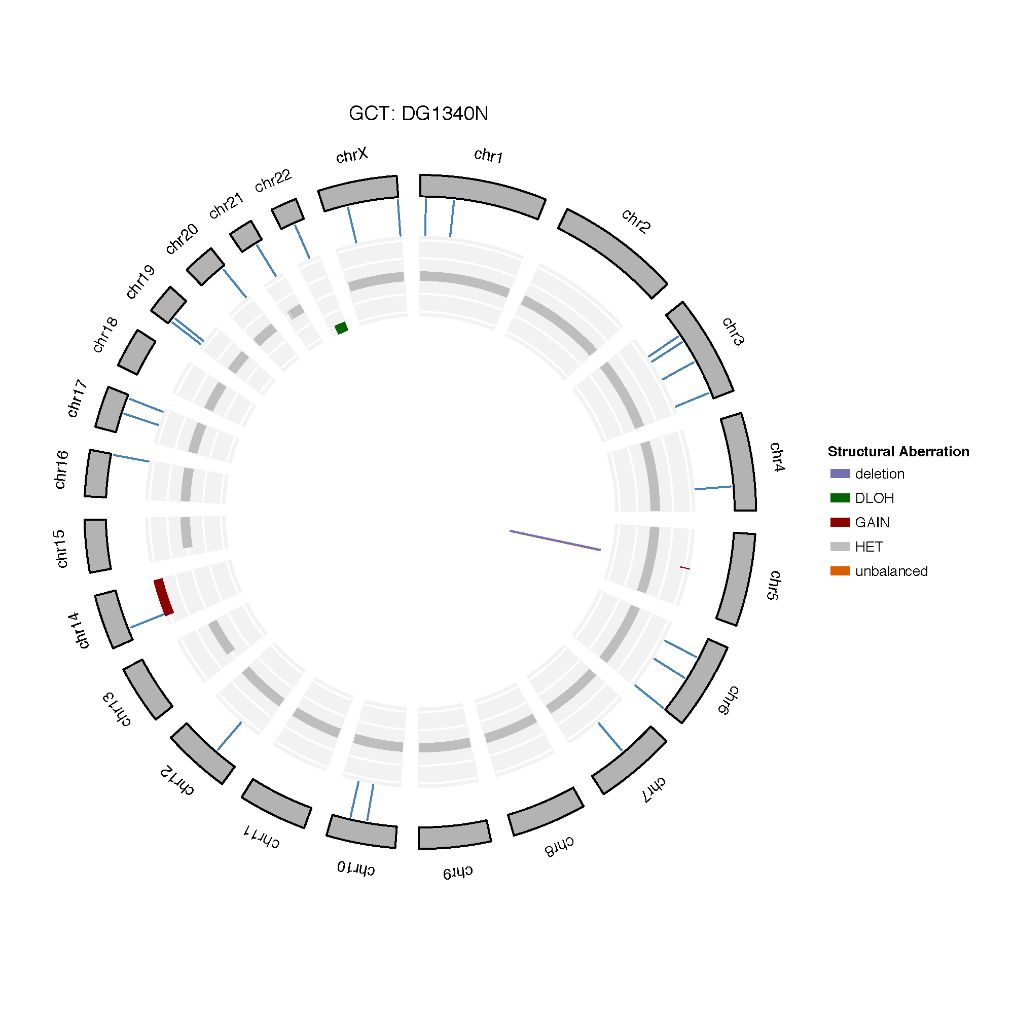
**

**Figure S2.** Circos plots of adult-type granulosa cell tumors from the whole genome sequencing exploratory cohort. The outermost track indicates the chromosome number. The next ring indicates the copy number information. The X chromosome is present in one copy. The lines in the interior of the circle illustrate chromosome regions involved in intra- or inter-chromosomal rearrangements. 

Abbreviations: ALOH – amplified loss of heterozygosity; ASCNA – allele-specific copy number amplification; DLOH – deletion loss of heterozygosity; GAIN – copy number gain; HET – diploid; NLOH – neutral loss of heterozygosity; UBCNA – unbalanced copy number amplification
